# Generation of NOD SCID mice with near-complete deletions of *Il2rg* and *Prkdc* for human cancer and HSC engraftment

**DOI:** 10.1007/s11248-025-00454-9

**Published:** 2025-07-11

**Authors:** You-Min Kim, Hee Ju Na, Do Hee Kwon, Jae Hoon Lee, Bo Min Park, Subin Lee, Tae Wook Nam, Mi Yeon Park, Sun Ha Park, Sung Joo Kim, Bongkum Choi, Han-Woong Lee

**Affiliations:** 1 Department of Biochemistry, College of Life Science and Biotechnology, Yonsei University, Seoul, 03722 Republic of Korea; 2 Transplantation Research Center, Research Institute for Future Medicine, Samsung Medical Center, Gangnam-gu, Seoul, 06351 Republic of Korea; 3 GEMCRO, Inc., Seoul, 03722 Republic of Korea; 4 Jeju Halla Hospital, Jeju-si, Jeju-do 63127 Republic of Korea; 5 Department of Health Science & Technology, Samsung Advanced Institute for Health Science & Technology (SAIHST), Sungkyunkwan University, Seoul, 06355 Republic of Korea

**Keywords:** Animal model, Immunodeficient mouse, Humanized mouse, Xenotransplantation, CRISPR/Cas9 system

## Abstract

Immunodeficient mouse models are invaluable tools for preclinical research, particularly for cancer therapies and studies of the human immune system. Notably, strains with combined *Prkdc* (scid) and *Il2rg* (null) mutations—such as NOG and NSG mice— are widely used due to their profound immunodeficiency, allowing efficient engraftment of various human cells. However, these models were generated by disrupting the *Il2rg* gene through replacement with a neomycin resistance (Neo) cassette in embryonic stem cells. Incomplete excision of this cassette can inadvertently alter the expression of neighboring genes, thereby introducing potential confounding variables. In addition, they may still express mutant mRNAs that escape nonsense-mediated decay (NMD) and/or produce truncated proteins with residual activity, potentially compromising the interpretation of experimental outcomes. To address this, we developed the N2G mouse strain (NOD-2-Genes KO) where almost all genomic loci of both *Prkdc* and *Il2rg* genes are deleted via CRISPR/Cas9 genome editing. N2G mice exhibited tumor growth comparable to NOG mice following the transplantation with several human cancer cell lines. Moreover, human CD34^+^ cord blood (CB) cells engrafted into N2G mice showed robust reconstitution of human immune cells, especially T cells in peripheral blood, spleen and bone marrow, compared to NSG mice. These results suggest that N2G mice, lacking residual mutant mRNA and the exogenous Neo resistant gene, offer an advanced model for preclinical studies.

**Supplementary Information:**

The online version contains supplementary material available at 10.1007/s11248-025-00454-9.

## Introduction

Humanized mouse models are indispensable tools in preclinical research, enabling the development of novel therapeutic strategies, the study of human cancers, the investigation of immune responses to pathogenic infections, and the understanding of immune cell maturations (Choi et al. 2011a, 2020). Since the first successful engraftment of human leukocytes and hematopoietic cells into mice (Mosier et al. 1988), various humanized mouse models have been developed to facilitate the reconstitution of human immune cells (Choi et al. 2011b, 2011a). Among these, NOD.Cg-*Prkdc*^*scid*^*Il2rg*^*tm1Wj*^ (NSG) and NOD.Cg*-Prkdc*^*scid*^*Il2rg*^*tm1Su*^ (NOG) mice are widely used due to their severe immunodeficiency, characterized by the absence of T cells, B cells, and natural killer (NK) cells, which enables stable engraftment and functional reconstitution of human cells. However, despite their widespread use, these models have inherent limitations that require further improvement (Shultz et al. 2019, 2012; Kenney et al. 2016; Hu et al. 2011). These limitations arise from the residual portions of the *Prkdc* and *Il2rg* genes that remain after conventional gene knockout approaches, which were developed either by replacing specific exons with a neomycin resistant gene cassette using embryonic stem (ES) cells or by inducing indel-mediated frameshifts using CRISPR/Cas9 technology. This can potentially lead to the production of truncated proteins or mutant mRNAs that may retain partial functionality and interfere with experimental outcomes (Park and Lee 2024; Lee et al. 2019). These unintended gene products could affect studies involving immune system interactions, cytokine signaling, or long-term engraftment efficiency.

The *Prkdc* scid mutation leads to the truncation of the catalytic subunit of DNA-dependent protein kinase (DNA-PKcs) at its C-terminus (Pearson et al. 2008; Mathieu et al. 2015). This truncation severely impairs DNA double-strand break repair, resulting in defective T and B lymphocyte development and causing combined immunodeficiency (SCID) (Mathieu et al. 2015; Lee et al. 2023). However, cells with the scid mutation continue to express the truncated DNA-PKcs protein, which retains the ability to bind DNA despite lacking kinase activity (Mathieu et al. 2015). The presence of this kinase-deficient, yet DNA-binding, protein can complicate experimental interpretations (Beamish et al. 2000), as its residual non-enzymatic functions may interfere with studies requiring complete immunodeficiency (Lee et al. 2023). Furthermore, immune system “leakiness” can occur in scid mice, occasionally allowing partial lymphocyte development, particularly with age, thereby introducing variability that challenges the consistency of preclinical research outcomes (Yasuda et al. 2017; Ito et al. 2002).

The *Il2rg* gene encodes the common gamma chain (γc), a shared receptor subunit essential for signaling through multiple cytokines, including interleukin (IL)-2, IL-4, IL-7, IL-9, IL-15, and IL-21 (Bak et al. 2020). These cytokines play critical roles in the development, survival, and activation of various immune cells, such as T cells, B cells, NK cells, and macrophages (Bak et al. 2020). Accordingly, loss of *Il2rg* function leads to complete absence of NK cells, enhancing the engraftment and long-term survival of human cells in immunodeficient models (Kenney et al. 2016).

Widely used NSG and NOG mice carry mutations in both *Prkdc* and *Il2rg*; among these, the *Il2rg* gene was disrupted in embryonic stem cells by replacing it with a selection marker, such as the neomycin resistance (Neo) cassette (Cao et al. 1995). However, in some cases, the Neo cassette is not properly excised after selection, and if retained, it can influence the expression of neighboring genes in the genome (Pham et al. 1996; Scacheri et al. 2001; Jin et al. 2020). Therefore, the possibility of unintended side effects due to residual Neo cassettes cannot be completely excluded in NSG and NOG mice. Moreover, current knockout approaches, often employing CRISPR/Cas9-mediated small deletions, can result in mutant mRNAs that escape nonsense-mediated mRNA decay and produce truncated proteins retaining partial or unknown functionality (Mou et al. 2017; Sui et al. 2018; Anderson et al. 2017; Lee et al. 2020). Such residual elements may compromise the experimental outcomes, especially in studies involving cytokine signaling or immune system interactions (Hong et al. 2014). These limitations highlight the need for more precise genetic engineering strategies that ensure complete functional null mutations of *Il2rg*, thereby enabling more reliable modeling of immunodeficient systems.

In this study, we addressed these challenges by developing a novel immunodeficient mouse model, N2G, using CRISPR/Cas9 technology to achieve almost complete deletions of both *Prkdc* and *Il2rg* genes. This strategy eliminates the expression of truncated or mutant proteins, providing a more reliable platform for preclinical research requiring robust and predictable immunodeficiency. We evaluated the efficacy of N2G mice in supporting human cancer cell xenograft growth and human immune cell reconstitution. We suggest that the N2G model offers an improved platform for studying human cancers and immune responses, potentially reducing discrepancies observed in current immunodeficient models.

## Materials and methods

### Generation of N2G mice

The N2G mice were generated by GEMCRO Inc. (Seoul, Korea) using Toolgen’s CRISPR/Cas9 technology on a NOD strain background as following standard procedures. For CRISPR/Cas9 system, dual guide RNAs consisting of a CRISPR RNA (crRNA) and a trans‐activating crRNA (tracrRNA) were utilized. These dual guide RNAs and Cas9 protein were micro-injected into fertilized eggs. Each crRNA specifically targeted exon 3 and exon 86 of *Prkdc* on chromosome 16, and the upstream region (promoter) of exon 1 and exon 8 of *Il2rg* on chromosome X. To design these guide RNAs, we used Cas-OFFinder (Bae et al. 2014) to minimize off-target potential and applied the Doench score (Doench et al. 2016) to maximize editing efficiency. The potential off-target mutations were stringently excluded through subsequent backcrossing. Only correctly edited alleles were maintained through successive backcrossing with NOD mice during the establishment of the N2G line. The target sequence of crRNA for *Prkdc* in exon 3 were 5′‐GAGAGTAATGCATAACCTTC‐3′ and 5′‐ACTCTCTTGATATTAAGGTA‐3′; in exon 86 were 5′‐GTTAGTTAGGCCATTAGCAT‐3′ and 5′‐TGGTTCACTGCCTCCAATAT‐3′. For *Il2rg* in promoter, the target sequences of crRNA were 5′‐CCTGAGGTTTCAAGTCGGGC‐3′ and 5′‐GATGATGCTATTTATTAAGC‐3′; in exon 8 were 5′‐GAAATCGAAACTTAGCCCCA‐3′ and 5′‐ACACTCTCAAGTAGGGCATA‐3′. Injected embryos were transplanted into the recipient pseudopregnant female mice. The deletion of *Prkdc* and *Il2rg* was identified by PCR analysis of genomic DNA isolated from the tail of the mouse using specific primers as follows: for *Prkdc*, a forward primer 5′-GGAAGTCGCTTAGCATTGAGGA-3′ and a reverse primer 5′-AGAATGCTTCTGCCTGATGATCT-3′; for *Il2rg*, a forward primer 5′-AAAGGGGACCAGTTTGTGGG-3′ and a reverse primer 5′-GAAAGGTGTTAGGCTGGGCA-3′ (Fig. S1A). The deletion in the selected mice was validated by sequencing of PCR product amplified from the targeted loci (Bionics, Korea). N2G mice were subsequently generated by crossbreeding mice carrying the *Prkdc* deletion with those carrying the *Il2rg* deletion.

#### Animals

All mice were housed in a specific pathogen-free (SPF) animal facility at the Yonsei Laboratory Animal Research Center. The mice were kept in a controlled environment with a 12-h light/dark cycle, provided with a normal chow diet, and given free access to food and water. All procedures were conducted in accordance with the Korean Ministry of Food and Drug Safety guidelines for animal research. The study was approved by the Institutional Animal Care and Use Committee of Yonsei University (approval number: IACUC-202111-1361-03).

#### RNA extraction, reverse transcription, and RT-qPCR

Total RNA was extracted from mouse tissue samples using the TRIzol (Ambion) according to the manufacturer’s instructions. Complementary DNA (cDNA) was synthesized from 1 µg of total RNA using the RevertAid First Strand cDNA Synthesis Kit (Thermo Scientific™), following the instructions of the manufacturer. The resulting cDNA was diluted 1:10 in nuclease-free water. RT-qPCR was performed with SYBR Green PCR Master Mix (Meridian Bioscience). Relative gene expression was calculated using *Gapdh* as the reference gene. RT-qPCR was conducted using CFX (Bio-Rad) system with the following primers: *Gapdh*, 5′-CATCACTGCCACCCAGAAGACTG-3′ (forward) and 5′-ATGCCAGTGAGCTTCCCGTTCAG-3′ (reverse); *Prkdc*, 5′-AAGGCAGAAGCCTGGACAAGTG-3′ (forward) and 5′-ATCCGCCAGTAGGTCAATGCTG-3′ (reverse); and *Il2rg*, 5′-TTCTACAGCCCCTGAACACCTCA-3′ (forward) and 5′-CCTTGTACCTATAGTGCAGCGTG-3′ (reverse).

#### Cells

The A549 and MDA-MB-231 cell lines were obtained from the American Type Culture Collection (ATCC; CCL-185 and HTB-26, respectively). A549 cells were cultured in Roswell Park Memorial Institute (RPMI) 1640 Medium (Welgene), while MDA-MB-231 cells were maintained in high-glucose Dulbecco’s Modified Eagle’s Medium (DMEM, Welgene), both supplemented with 10% fetal bovine serum (FBS) and 100 units/ml penicillin/streptomycin (Gibco). The cells were maintained in a humidified incubator at 37 °C with 5% CO_2_.

#### Cell line-derived xenograft model

Subcutaneous injections of various human cancer cell lines were performed to evaluate tumor engraftment in N2G and NOG mice. Specifically, 1 × 10^6^ A549 cells suspended in 50 μl of PBS were bilaterally injected into the flanks of 6–8-week-old male N2G and NOG mice. In a separate cohort, 1 × 10^6^ MDA-MB-231 cells in 50 μl PBS were similarly administered into both flanks of female N2G mice. For a high-dose engraftment study, 5 × 10^6^ A549 cells in 50 μl PBS were injected into the right flank of 10-week-old male N2G mice. Additionally, 8 × 10^6^ SK-BR3 cells in 100 μl PBS were delivered subcutaneously into the right flank of 8-week-old female N2G mice. To establish an orthotopic breast cancer model, 5 × 10^6^ MDA-MB-468 cells in 100 μl PBS were injected into the mammary fat pad of 7-week-old female N2G mice. Tumor size was measured once a week after the implantation. Tumor volume was calculated using the following formula:

$${\text{Tumor}}\;{\text{volume}}\;({\text{mm}}^{3} ) = {\text{Length}}\;({\text{mm}}) \times {\text{Width}}\;({\text{mm}})^{2} \times 0.5$$

At the end of the study, when the tumor volume reached over 1000 mm^3^, all animals were euthanized by cervical dislocation and subjected to necropsy.

#### Isolation of human CD34^+^ (hCD34^+^) cells from cord blood

The human protocol for the experiments with human materials was approved by the Institutional Review Boards of Samsung Medical Center (SMC), Seoul, South Korea (IRB No.: 2010-09-060). Human cord blood (hCB) samples were acquired from full-term deliveries after obtaining informed parental consent according to guidelines established by the SMC. Mononuclear cells (MNCs) were isolated using Ficoll-Hypaque density gradient centrifugation. Human CD34^+^ (hCD34^+^) cells were subsequently purified by positive selection using the MACS human CD34 MicroBead Kit (Miltenyi Biotec) and autoMACS™ Pro Separator (Miltenyi Biotec), according to the manufacturer’s instructions. The purified hCD34^+^ cells were analyzed by flow cytometry analysis using antibodies specific for hLin (anti-human lineage cocktail 1, BD), hCD34 (BD Pharmingen™), and hCD38 (eBioscience). Only hLin^−^CD34^+^CD38^−^ cells with a purity of over 95% were used in the study.

#### Generation of humanized mice

Six- to eight-week-old N2G and NSG mice were preconditioned with busulfan prior to transplantation of hCD34^+^ cells. Busulfan (Buselfex, Otsuka) was purchased at a concentration of 6 mg/mL and diluted with 0.9% saline before use. The mice were intraperitoneally injected with 250 μl of busulfan solution at doses of 20, 25, or 30 mg/kg body weight. Twenty-four hours later, hCD34^+^ cells (1.5 × 10^5^ in 100 μl PBS), purified from hCB, were intravenously transplanted into the busulfan-pretreated mice via the tail vein.

### Flow cytometric analysis

After hCD34^+^ CB cell transplantation, peripheral blood (PB) was collected from the intraorbital vein at 4-week intervals and treated with 1 × RBC Lysis Buffer (Invitrogen) according to the manufacturer’s instruction. At 24 weeks post-transplantation, all humanized N2G (hu-N2G) and humanized NSG (hu-NSG) mice were sacrificed, and the spleen, bone marrow, and PB cells were collected. Cells were prepared from the samples and suspended in PBS (Cytiva). For flow cytometric analysis, the following human-specific monoclonal antibodies were used: anti-CD16-BV421 (Clone 3G8, BioLegend), anti-CD3-eFluor450 (Clone UCHT1, Invitrogen), anti-CD4-BV570 (Clone RPA-T4, BioLegend), anti-CD14-SuperBright600 (Clone 61D3, Invitrogen), anti-CD19-SuperBright645 (Clone HIB19, Invitrogen), anti-CD8-SuperBright702 (Clone SK1, Invitrogen), anti-CD56-SuperBright780 (Clone TULY56, Invitrogen), anti-CD11c-FITC (Clone 3.9, Invitrogen), anti-CD33-PE (Clone WM-53, Invitrogen), anti-CD15-PE-Dazzle594 (Clone W6D3, BioLegend), anti-CD123-PE-Cy5 (Clone 6H6, Invitrogen), anti-CD11b-PerCP-eFluor710 (Clone ICRF44, Invitrogen), anti-CD68-PE-Cy7 (Clone eBioY1/82A, Invitrogen), anti-CD66b-APC (Clone G10F5, Invitrogen), anti-CD45-Alexa-Fluor700 (Clone 2D1, Invitrogen), and anti-HLA-DR-APC-H7 (Clone G46-6, BD). The cells (1 × 10^6^) were stained with appropriate antibodies in 100 μl of PBS. Stained cells were analyzed on Cytek Northern Lights (Cytek Biosciences). Ten thousand to one million events were acquired per sample and analyzed using the FlowJo flow cytometry analysis program (BD).

#### Cytokine stimulation response assay

Splenocytes were isolated from hu-N2G and hu-NSG mice at 24 weeks post-transplantation and treated with 1X RBC Lysis Buffer (Invitrogen) according to the manufacturer’s instructions. The cells were then suspended in RPMI 1640 medium (Welgene) and treated with 5 μg/ml IL-2, 25 μg/ml IL-18, and 50 ng/ml IL-15 for 1 h. After cytokine treatment, GolgiSTOP (BD Biosciences) was added to halt activation. The treated cells were cultured in a 96-well plate containing 1 × 10^6^ cells/well and incubated at 37 °C in CO_2_ incubator for 24 h. Following incubation, the cells were stained with the following human-specific monoclonal antibodies: anti-CD3-eFluor450 (Clone UCHT1, Invitrogen), anti-CD4-BV570 (Clone RPA-T4, BioLegend), anti-CD8-SuperBright702 (Clone SK1, Invitrogen), anti-CD56-SuperBright780 (Clone TULY56, Invitrogen), and anti-CD45-Alexa-Fluor700 (Clone 2D1, Invitrogen). Surface-stained cells were permeabilized using the CytoFix/CytoPerm Kit (BD Biosciences), and intracellular cytokines were stained with anti-IFN-γ-BV605 (Clone B27, BD), and anti-TNF-α-PE-Cy7 (Clone MAb11, BD). Cells were analyzed using a Cytek Northern Lights (Cytek Biosciences), and the results were analyzed by the FlowJo flow cytometry analysis program (BD Biosciences).

#### Cell Proliferation ability assay

The splenocytes were isolated and treated with 1 × RBC Lysis Buffer (Invitrogen) according to the manufacturer’s protocol. Cells were then suspended in PBS (Cytiva) and labeled using the CellTrace CFSE Cell Proliferation Kit (Invitrogen) following the manufacturer’s instructions. The labeled cells were treated with anti-CD3 and anti-CD28 T-Activator (50ul/ml, Invitrogen) according to the manufacturer’s protocol. Stained cells were cultured in a 96-well plate containing 1 × 10^6^ cells/well, and incubated in a 37 °C CO_2_ incubator for 0 to 3 days. After incubation, cells were harvested daily and analyzed by flow cytometry (Cytek Biosciences) using a 488 nm emission wavelength.

#### Histology

At the end of this study, spleens from hu-N2G and hu-NSG mice were fixed in 10% formalin and embedded in paraffin. The paraffin-embedded tissues were sliced into 4 μm sections and subjected to standard hematoxylin and eosin (H&E) staining or immunohistochemistry (IHC). IHC was performed using the Peroxidase/DAB Rabbit/Mouse (DAKO) kit according to the manufacturer’s protocols. Nuclei were counterstained with hematoxylin. Primary antibodies used for IHC were rabbit polyclonal anti-hCD3 (1:1000, Dako), mouse monoclonal anti-hCD20 (1:50, abcam), and mouse monoclonal anti-hCD68 (1:100, abcam). Stained slides were observed using an Olympus CX41 light microscope (Olympus) with a 200 × numerical aperture objective, and photographic images were collected with Vitual Slide System (Aperio Technologies) and analyzed using Aperio ImageScope software (Aperio Technologies).

### Statistical analysis

All statistical analyses were performed using GraphPad Prism 6 (GraphPad Software). A *p*-value of less than 0.05 was considered statistically significant (*p < 0.05, **p < 0.01, ***p < 0.001, ****p < 0.0001).

## Results

### Generation of N2G mice with extensive deletion of *Prkdc* and complete deletion of *Il2rg* genes

**Fig. 1:**
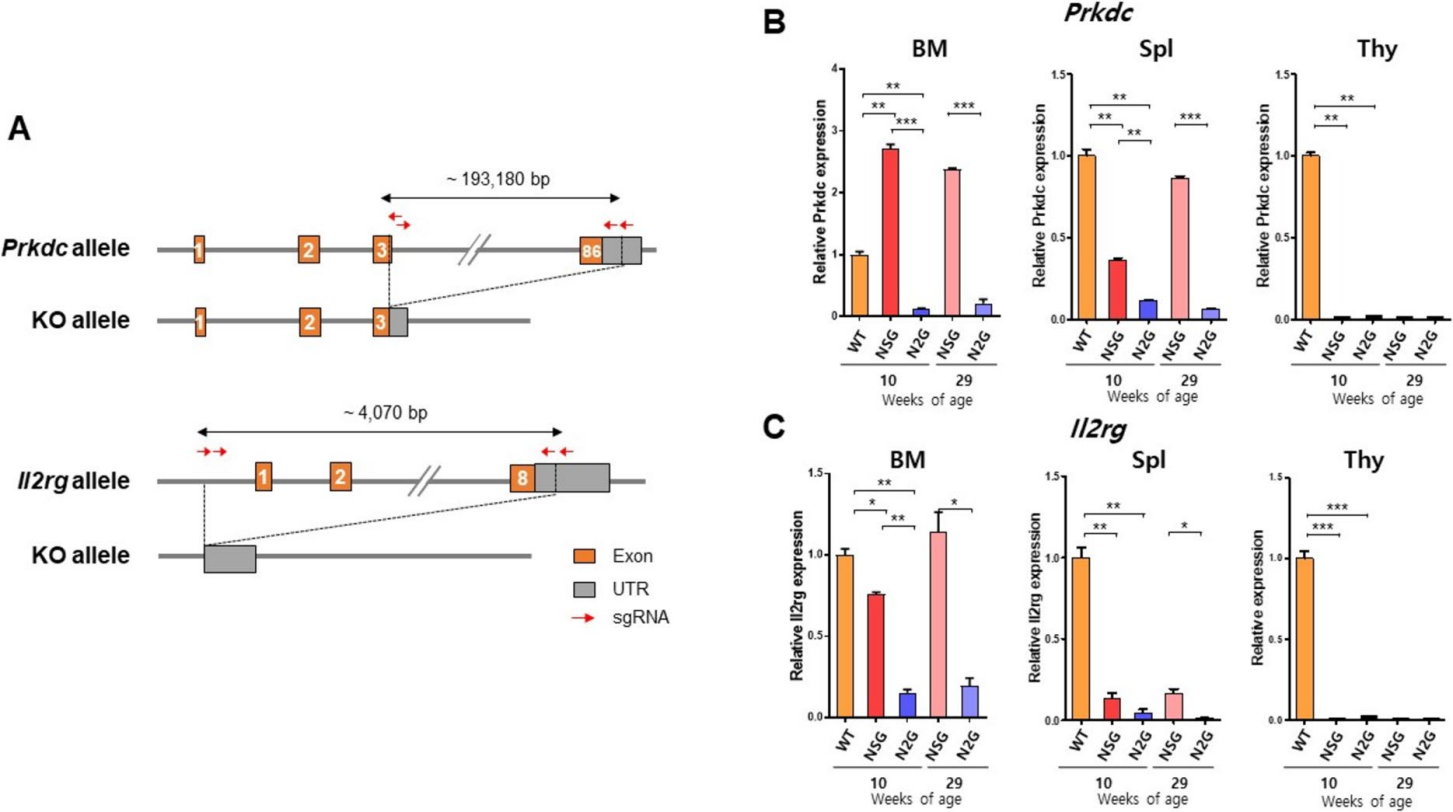
Generation of N2G mouse model by CRISPR/Cas9 system. **A** A schematic diagram illustrating gRNAs target sites in *Prkdc* and *Il2rg* loci in the mouse genome. CRISPR-Cas9-mediated genome editing results in the deletion of exons 3 to the 3’ UTR of the *Prkdc* gene, and exons 1 to the 3’ UTR of the *Il2rg* gene. **B** and **C** Relative mRNA expression of (**B**) *Prkdc* and (**C**) *Il2rg* in bone marrow (BM), spleen (Spl), and thymus (Thy) of 10- and 29-week-old N2G (*n* = 2) and NSG (*n* = 2) mice, assessed by RT-qPCR using *Gapdh* as the reference gene. Ten-week-old BALB/c (*n* = 2) mice were used as WT controls. Data are presented as mean ± standard deviation (SD)

To eliminate residual regions of the *Prkdc* and *Il2rg* genes present in existing NOD/scid/*Il2rg*^null^ mice—traditionally generated via embryonic stem (ES) cell-based gene targeting or CRISPR/Cas9-mediated indels—we employed an advanced CRISPR/Cas9 genome editing strategy using multiple guide RNAs (gRNAs) to achieve comprehensive deletion of both genes in NOD mice. For *Prkdc*, gRNAs were designed to target exon 3 and the last exon (exon 86), leading to the removal of a 193,180-bp genomic region spanning exon 3 to 86 (Fig. 1A). Similarly, for *Il2rg*, gRNAs targeted the promoter region and the last exon (exon 8), successfully deleting a 4,070-bp genomic region from exon 1 to 8 (Fig. 1A). This approach ensures the nearly complete loss of *Prkdc* and *Il2rg* genomic DNA, thereby enhancing the reliability and utility of NOD-derived immunodeficient models in preclinical research.

To confirm the genomic deletion of the targeted loci, we performed PCR-based genotyping using primers flanking the deleted regions of *Prkdc* and *Il2rg*. Agarose gel electrophoresis of PCR amplicons revealed distinct band shifts between N2G and wild-type mice, consistent with the expected size differences caused by the CRISPR/Cas9-mediated deletions. These results support the successful excision of the targeted genomic regions at the DNA level (Fig. S1A).

To evaluate the efficacy of *Prkdc* and *Il2rg* gene deletions in N2G mice, we measured mRNA expression levels using RT-qPCR. In N2G mice, aged 10 and 29 weeks, *Prkdc* and *Il2rg* mRNAs were undetectable in the bone marrow, spleen and thymus, whereas robust expression was observed in wild-type (WT) mice (Fig. 1B, C). Notably, *Prkdc* and *Il2rg* mRNA levels were obviously detectable in the bone marrow and spleen of NSG mice, with an age-dependent increase. This phenomenon is consistent with previously reported immune system “leakiness” in NSG mice, where partial development of lymphocytes can occur with aging (Yasuda et al. 2017; Ito et al. 2002). In contrast, N2G mice showed minimal or undetectable expression of these genes even at 29 weeks of age (Fig. 1B, C), indicating more robust immunodeficiency and reduced leakiness in this model. Moreover, NSG mice even demonstrated comparable or higher mRNA levels of *Prkdc* or *Il2rg* than WT mice (Fig. 1B, C). These findings indicate the successful generation of N2G mice with substantial reduction of *Prkdc* and *Il2rg* expression across multiple tissues, including complete ablation in the thymus and minimal residual expression in the spleen and bone marrow. This comprehensive deletion eliminates the almost entire coding regions, thereby completely removing the possibility of mutant mRNAs or truncated proteins expression.

### Tumor engraftment in N2G mice

**Fig. 2:**
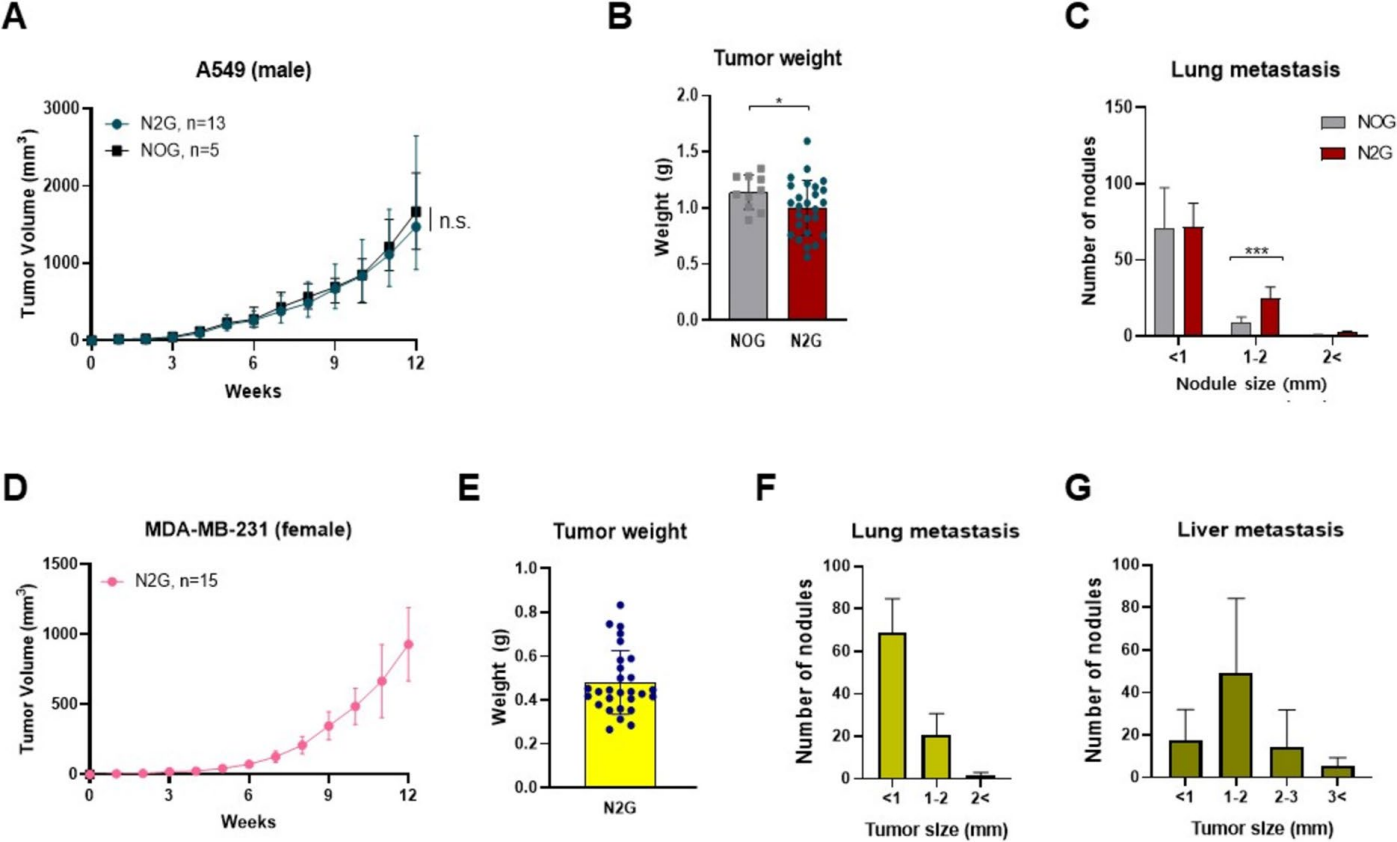
Cell line-derived xenograft in N2G mice. **A** and **B** Tumor volumes (**A**) and tumor weights (**B**) after subcutaneous injection of 1 × 10^6^ A549 lung cancer cells into 8-week-old male N2G (*n* = 13) and NOG (*n* = 5) mice. **C** Number of metastatic lung tumor nodules in N2G and NOG mice, counted based on nodule size. **D** and **E** Tumor volumes (**D**) and tumor weights (**E**) after subcutaneous injection of 1 × 10^6^ MDA-MB-231 breast cancer cells into 15-week-old female N2G (*n* = 15) mice. Tumor volume was monitored weekly, and tumor weight was measured at 12 weeks post-xenograft. **F and G** Number of metastatic nodules in the (**F**) lungs and (**G**) liver of N2G mice, counted based on nodule size. Data are presented as mean ± S.D. Statistical significance: *p < 0.05, ***p < 0.001

Immunodeficient mice, which lack the ability to reject human xenografts, are essential for preclinical cancer research (Sartelet et al. 2012). To evaluate the tumor-supporting potential of N2G mice, we conducted xenograft experiments using human cancer cell lines and compared their tumor growth to those in NOG mice, which were generated via ES cell-based targeting. A549 lung cancer cells were subcutaneously inoculated into both N2G and NOG mice (Fig. 2A–C). Tumors developed in all mice within two weeks post-inoculation, with comparable growth observed between strains (Fig. 2A). At 12-weeks endpoint, measurements of tumor volume and weight showed no statistically significant differences between N2G and NOG mice (Fig. 2B and Fig. S2A). Notably, A549-transplanted N2G mice exhibited a greater number and larger size of lung metastatic nodules compared to those observed in NOG mice (Fig. 2C and Fig. S2B). While the number of nodules smaller than 1 mm was comparable between N2G and NOG mice, nodules measuring 1–2 mm were significantly more frequent in N2G mice, with an average of approximately 25 nodules per mouse compared to 9 in NOG mice, a difference that was statistically significant (*p* < 0.001) (Fig. 2C and Fig. S2B). To further assess the metastatic potential of N2G mice, MDA-MB-231 breast cancer cells were transplanted into N2G mice, leading to robust tumor growth with consistent metastasis to the lung and liver (Fig. 2D–G and Fig. S2C, D). To further assess the tumorigenic potential of N2G mice, we subcutaneously transplanted a higher number of A549 lung cancer cells (5 × 10^6^ cells) into 10-week-old male N2G mice (Fig. S2E). As expected, tumor formation was observed more rapidly compared to standard-dose transplants (1 × 10^6^ cells). To evaluate the versatility of the N2G mice across different cancer types. SK-BR-3 cells (8 × 10^6^) were subcutaneously injected into the right flank of 8-week-old female N2G mice, and MDA-MB-468 cells (5 × 10^6^) were orthotopically introduced into the mammary fat pad of 7-week-old female N2G mice (Fig. S2F and G). In both cases, robust tumor formation was observed, demonstrating that the N2G model effectively supports the engraftment of diverse human cancer cell types, including HER2-positive and triple-negative breast cancer cells. These results demonstrate that N2G mice are suitable model for human cancer xenografts and metastasis studies.

### Generation of humanized N2G (hu-N2G) mice with human CD34^+^ CB cell transplantation

**Fig. 3:**
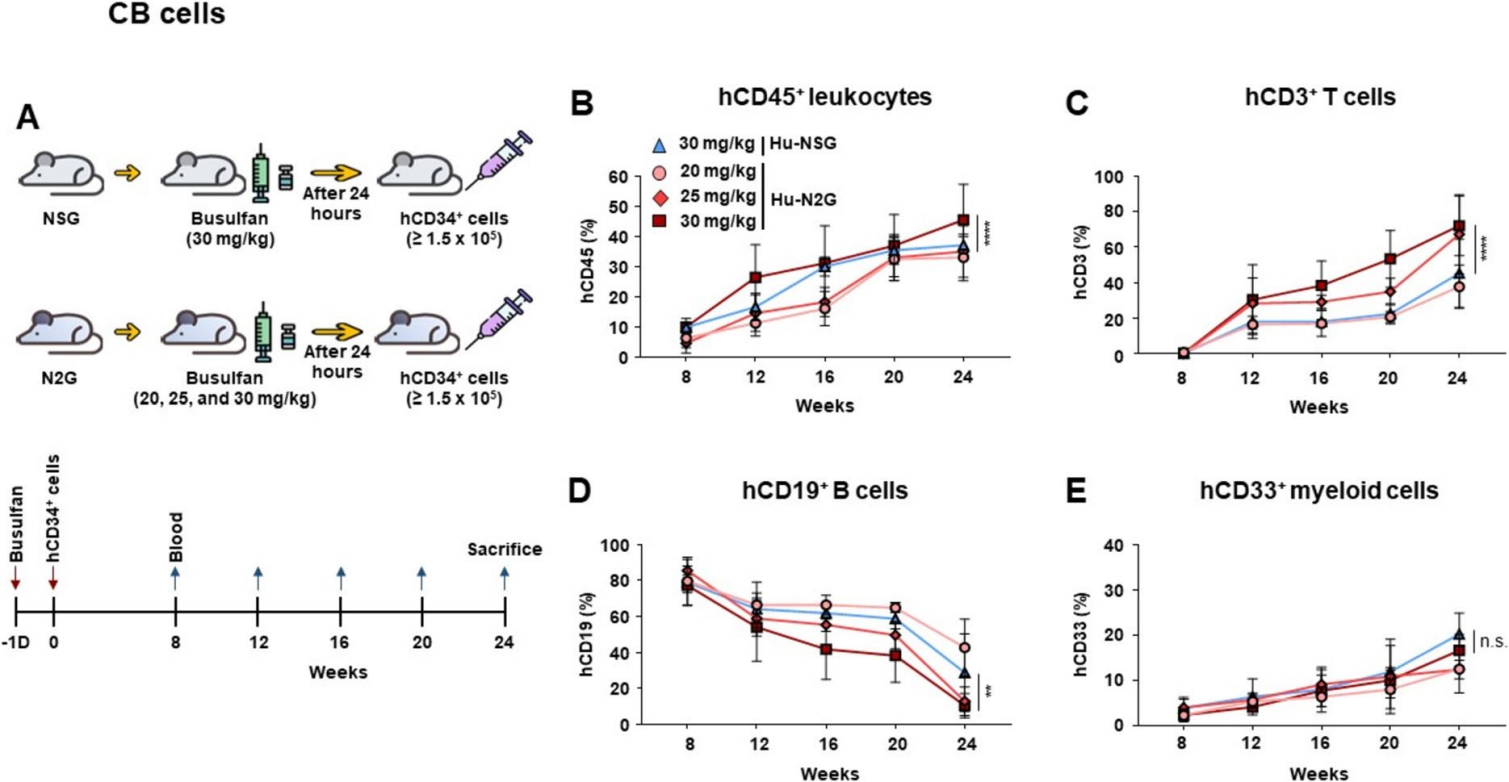
Generation of hu-N2G mice and reconstitution of human cells in peripheral blood with human CD34^+^ CB cells. **A** Schematic illustration showing the generation of hu-N2G (*n* = 10 per group) and hu-NSG (*n* = 5) mice. Six- to eight-week-old mice were intraperitoneally injected with busulfan at different doses (20, 25, and 30 mg/kg body weight). After 24 h, hCD34^+^ CB cells were intravenously injected into the busulfan-preconditioned N2G and NSG mice. D, Day. **B**–**E** Peripheral blood samples were collected from hu-N2G and hu-NSG mice at 4 weeks and at the indicated time points. The samples were stained with **B** anti-hCD45, **C** anti-hCD3, **D** anti-hCD19, and **E** anti-hCD33 antibodies and analyzed by flow cytometry. Data are presented as mean ± S.D. Statistical significance: *p < 0.05, **p < 0.01, ***p < 0.001

Immunodeficient mice are valuable for preclinical cancer research and also studies involving the human immune system (Choi et al. 2020; Shultz et al. 2012). Although it is not essential, preconditioning can substantially enhance the efficiency of human HSC transplantation in models aiming for robust immune cell reconstitution (Ishikawa et al. 2005). Whereas total body irradiation (TBI) has been commonly used (Sartelet et al. 2012; Shultz et al. 2005), its associated toxicity has prompted the exploration of alternatives such as busulfan conditioning, which offers a more convenient and less toxic approach (Ciurea and Andersson 2009; Choi et al. 2011a; Hayakawa et al. 2009). To identify an optimal preconditioning dose for human HSC engraftment, N2G mice were administered busulfan at doses of 20, 25, or 30 mg/kg, based on previously established dosing regimens in NSG models (Choi et al. 2011a; Hayakawa et al. 2009). For comparison, NSG mice received the standard 30 mg/kg dose (Choi et al. 2011a; Hayakawa et al. 2009) (Fig. 3A). Following transplantation of hCD34^+^ CB cells, human immune cell engraftment was monitored via flow cytometry every four weeks (Fig. 3A). The frequencies of hCD45^+^ leukocytes progressively increased in both N2G and NSG strains over time (Fig. 3B, C and Fig. S3A, B). Notably, humanizing N2G mice conditioned with 30 mg/kg busulfan (hu-N2G-bu30s) exhibited a stronger expansion of human CD3^+^ T cells compared to hu-NSG mice receiving the same dose (Fig. 3C). The frequency of hCD19^+^ B cells consistently declined over 24 weeks across all groups (Fig. 3D and Fig. S3C). hCD33^+^ myeloid cells gradually increased in all groups over 24-week period (Fig. 3E and Fig. S3D). Reconstitution analysis revealed that a 30 mg/kg busulfan conditioning in N2G mice resulted in higher frequencies of hCD45^+^ leukocytes, hCD3^+^ T cells, and hCD33^+^ myeloid cells, while a 20 mg/kg dose favored hCD19^+^ B cell engraftment (Fig. 3B–E). These findings confirm that N2G mice efficiently support human immune system development. The differential engraftment patterns observed with varying busulfan doses provide valuable insights for optimizing conditioning regimens in humanized mouse models.

**Fig. 4:**
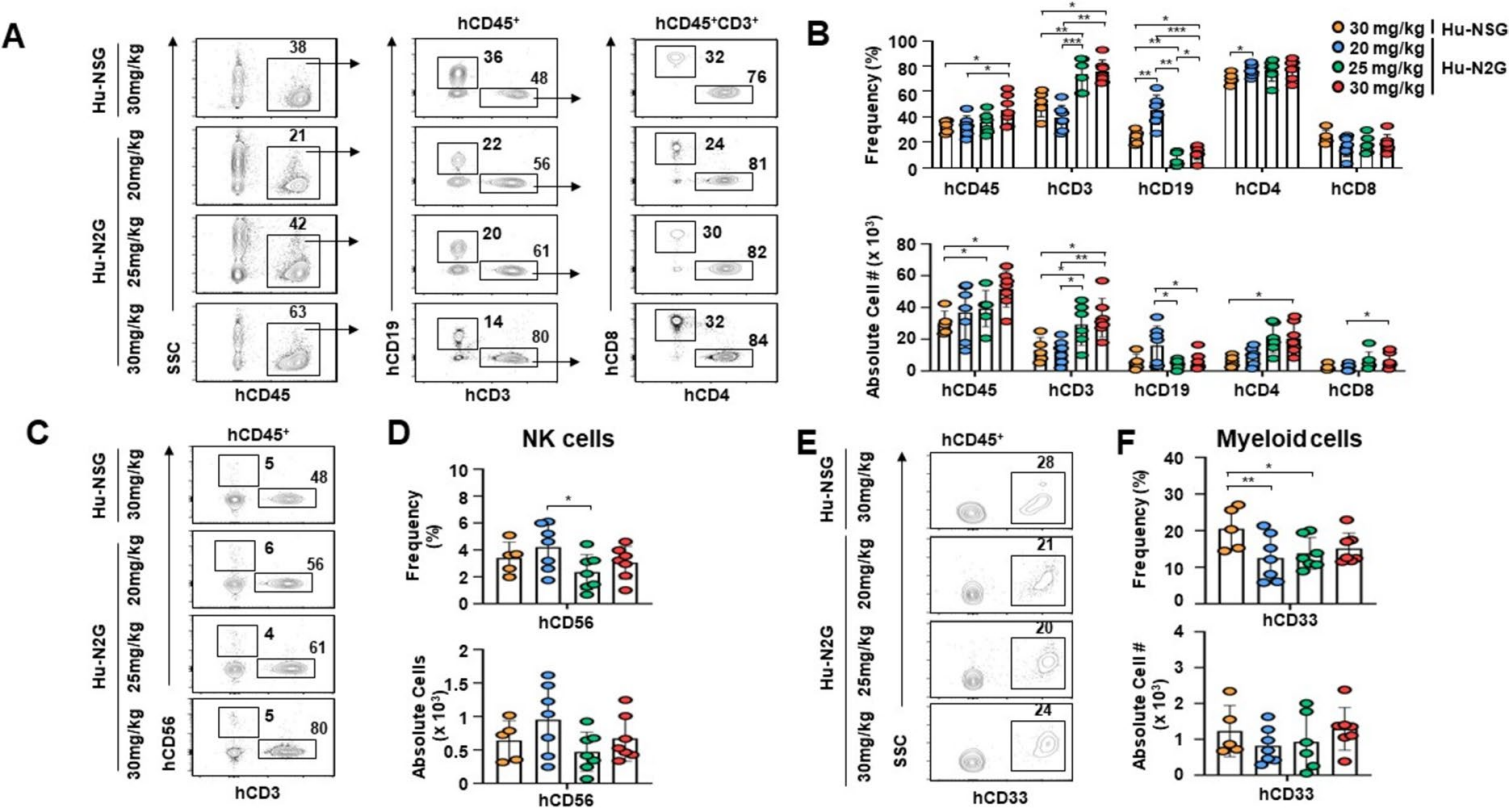
Reconstitution of human cells in peripheral blood of humanized mice. **A** Peripheral blood cells were isolated from the hu-N2G (*n* = 7, each group) and hu-NSG (*n* = 5) mice at 4 weeks after injection of hCD34^+^ CB cells. The cells were stained with anti-hCD45, anti-hCD3, anti-hCD19, anti-hCD4, and anti-hCD8 antibodies, followed by flow cytometry analyses. **B** Frequencies and absolute cell numbers were determined through flow cytometric gating and quantification. **C** Cells were stained with anti-hCD45, anti-hCD3, and anti-hCD56 antibodies followed by flow cytometry analyses. **D** Frequencies and absolute cell numbers were quantified using flow cytometric gating. **E** Cells were stained with anti-hCD45 and anti-hCD33 antibodies followed by flow cytometry analyses. **F** Frequencies and absolute cell numbers were obtained by flow cytometric gating and quantification. Data are presented as the mean ± S.D. Statistical significance: *p < 0.05, **p < 0.01, ***p < 0.001

To further examine the human immune cell reconstitution in humanized N2G mice conditioned with 30 mg busulfan (hu-N2G-bu30s), we performed flow cytometric analysis on peripheral blood and spleen samples. Hu-N2G-bu30s exhibited the highest frequency of hCD45^+^ leukocytes and hCD3^+^ T cells, whereas hCD19^+^ B cells were comparable to those in hu-NSG-bu30s (Fig. 4A, B). Similarly, the frequencies of hCD4^+^ T cells and hCD8^+^ T cells were also marginally higher in the hu-N2G-bu30s compared to other groups (Fig. 4A, B). The levels of hCD56^+^ NK cells were comparable across all groups (Fig. 4C, D). While the frequency of hCD33⁺ myeloid cells showed significantly increase in hu-NSG-bu30s among groups, the highest absolute counts were observed in hu-N2G-bu30s, with a modest increase in hu-NSG-bu30s. (Fig. 4E, F). Reconstitution of hCD34^+^ CB cells into monocytes and dendritic cells appeared to differ between hu-N2G-bu30s and hu-NSG-bu30s (Fig. S4A-D). However, their absolute numbers remained low (< 1000 cells), making these differences statistically insignificant (Fig. S4B, D). These results indicate that hu-N2G-bu30s retain human T cells more effectively than hu-NSG-bu30s, while showing comparable levels to other immune cell subsets in the peripheral blood.

**Fig. 5:**
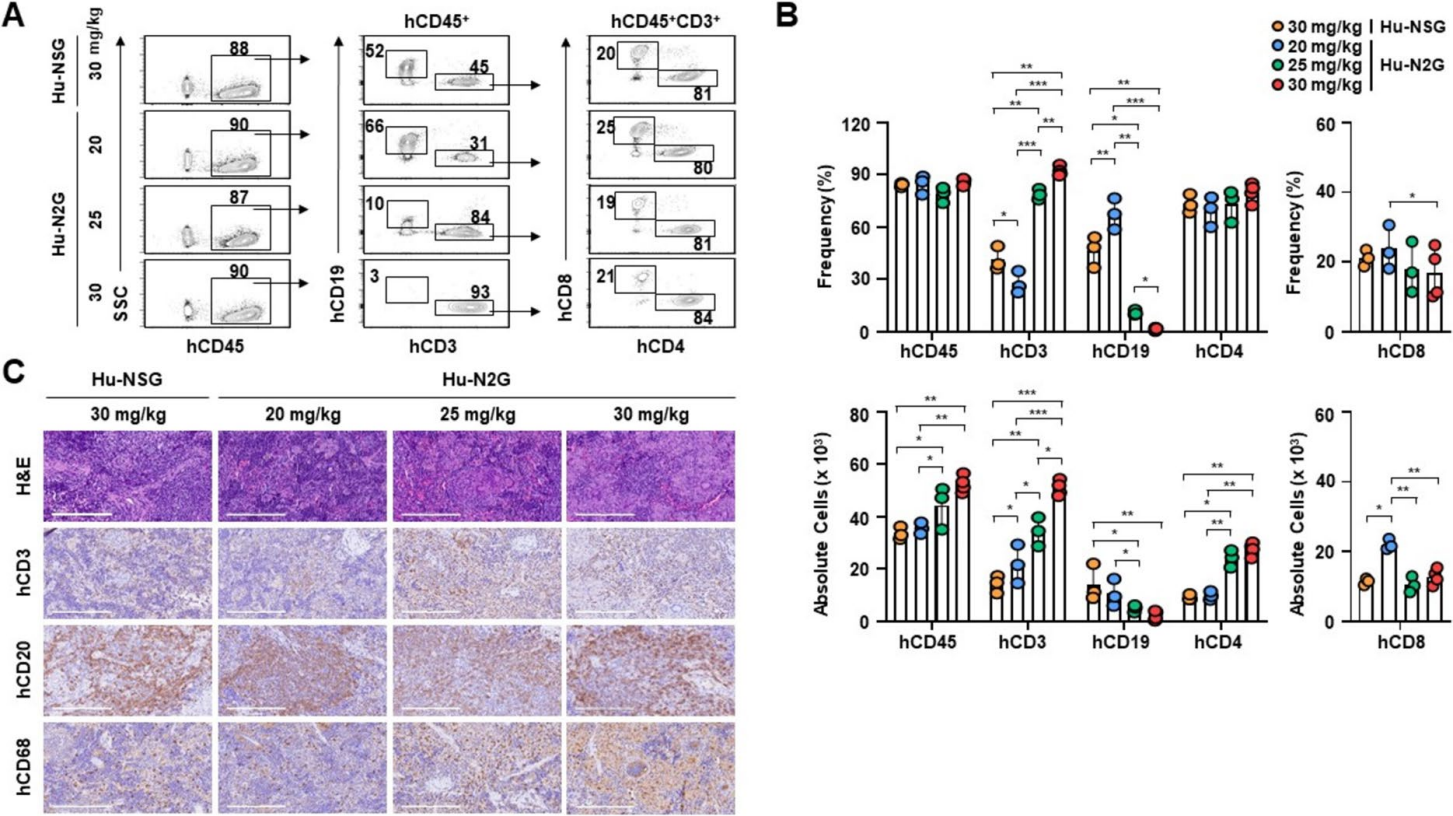
Reconstitution of human T and B cells in the spleen of humanized mice. **A** Splenocytes were isolated from the spleens of hu-N2G (*n* = 7, each group) and hu-NSG (*n* = 5) mice at 4 weeks after injection of hCD34^+^ CB cells. Cells were stained with anti-hCD45, anti-hCD3, anti-hCD19, anti-hCD4, and anti-hCD8 antibodies, followed by flow cytometry analysis. **B** Frequencies and absolute cell numbers were determined through flow cytometric gating. **C** Spleen tissues from hu-N2G and hu-NSG mice were performed H&E staining and immunohistochemistry with anti-hCD3, anti-hCD20, and anti-hCD68 antibodies. Stained samples were examined using an Olympus CX41 light microscope, and images were captured with a microscope digital camera DP50 and analyzed using an Image-pro Plus software. Data are presented as mean ± S.D. for each group of mice. Statistical significance: *p < 0.05, **p < 0.01, ***p < 0.001

Similarly, the frequency of hCD45^+^ leukocytes in the spleen was comparable across all groups, whereas the highest cell counts were observed in hu-N2G-bu30 (Fig. 5A, B). hCD3^+^ T cells were remarkably elevated in hu-N2G mice with increasing busulfan doses, compared to hu-NSG-bu30s (Fig. 5A, B). Although the frequencies of hCD4^+^ and hCD8^+^ T cells were similar among all groups, hu-N2G-bu30s exhibited the highest absolute number of hCD4^+^ T cells, whereas hu-N2G mice treated with 20 mg/kg busulfan (hu-N2G-bu20s) displayed the highest absolute number of hCD8^+^ T cells (Fig. 5A, B). These data indicate that N2G mice effectively reconstitute human T cells in the spleen, with comparable reconstitution of B cells, NK cells, and myeloid cells to hu-NSG mice.

Histological analyses further confirmed the presence of human immune cells in the spleens of both hu-NSG and hu-N2G mice. Notably, in these tissues, hCD3^+^ T cells and hCD19^+^ B cells were observed, along with hCD68^+^ human macrophages/monocytes (Fig. 5C). The staining intensity of hCD3^+^ T cells and hCD68^+^ macrophages was particularly strong in hu-N2G mice treated with 25 mg/kg busulfan (hu-N2G-bu25s) and hu-N2G-bu30s compared to other groups (Fig. 5C). Additionally, hCD20^+^ B cells were most prominent in hu-N2G-bu20s (Fig. 5C). These observations indicate that 30 mg/kg busulfan conditioning optimizes human immune cell reconstitution in the spleen. Furthermore, hu-N2G-bu30s also exhibited enhanced reconstitution into hCD19^+^ in the bone marrow compared to hu-NSG-bu30s, whereas hu-N2G-bu20s displayed the highest absolute number of hCD3^+^ T, hCD4^+^ and hCD8^+^ T cells (Fig. S5A, B). Collectively, these findings demonstrate that hu-N2G mice support efficient reconstitution of human T cells, B cells, NK cells, and macrophages/monocytes in peripheral blood, spleen, and bone marrow.

### Cytokine response of splenic human cytotoxic immune cells in humanized N2G mice

**Fig. 6:**
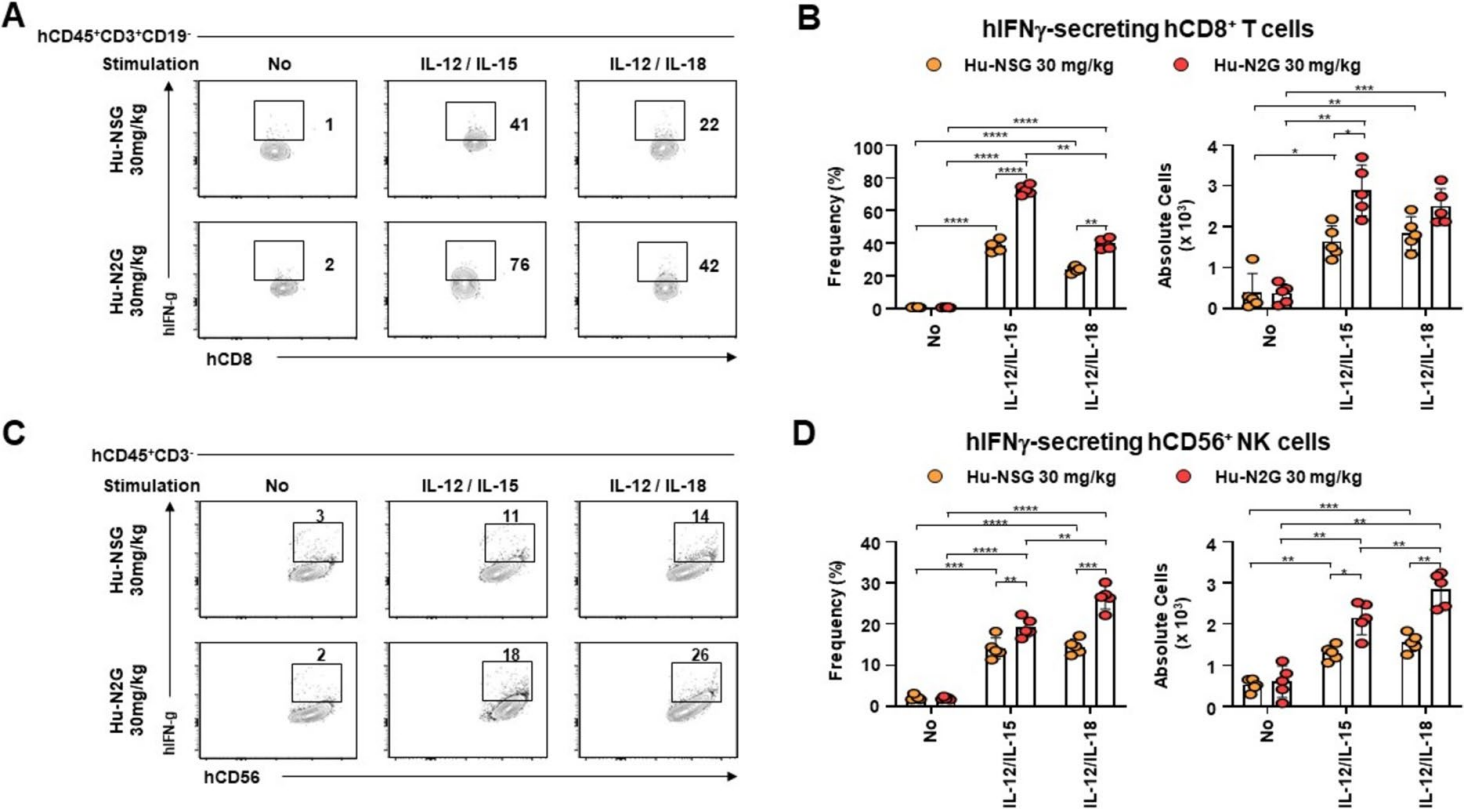
Cytokine stimulation response study from splenocytes. **A** and **C** Splenocytes were isolated from hu-N2G (*n* = 7, each group) and hu-NSG (*n* = 5) mice and treated with cytokines IL-12, IL-15, and IL-18. The cells were then stained with anti-hCD8, anti-hCD56, and anti-hIFN-γ antibodies. The hCD8^+^hIFN-γ^+^ **A** and hDC56^+^hIFN-γ^+^ **C** cells were identified using flow cytometry. **B** and **D** Frequencies and absolute cell numbers were determined by flow cytometric gating. Data are presented as mean ± S.D. Statistical significance: *p < 0.05, **p < 0.01, ***p < 0.001

Cytokines IL-12, IL-15, and IL-18 enhance the cytotoxic activity of T and NK cells and are commonly used to evaluate their functional responsiveness ex vivo (Smeltz 2007, French et al. 2006). To assess cytokine responsiveness in hu-N2G mice, splenocytes from hu-NSG and hu-N2G mice were stimulated with IL-12/IL-15 or IL-15/IL-18. Upon these stimulation, hIFNγ secretion was significantly increased in hCD8^+^ T cells and hCD56^+^ NK cells. Remarkably, hu-N2G mice exhibited significantly higher levels of hCD8^+^IFNγ^+^ T cells following cytokine stimulation compared to hu-NSG mice and unstimulated controls (Fig. 6A, B). Additionally, stimulation with IL-12/IL-15 or IL-12/IL-18 considerably elevated hIFNγ secretion in NK cells from hu-N2G mice (Fig. 6C, D). Collectively, these results suggest that human T and NK cells reconstituted in hu-N2G mice exhibit enhanced functional responsiveness to cytokine stimulation, highlighting the utility of this model for studying human cell-mediated immunity.

### Proliferation of splenic human T and NK cells of humanized N2G mice

**Fig. 7:**
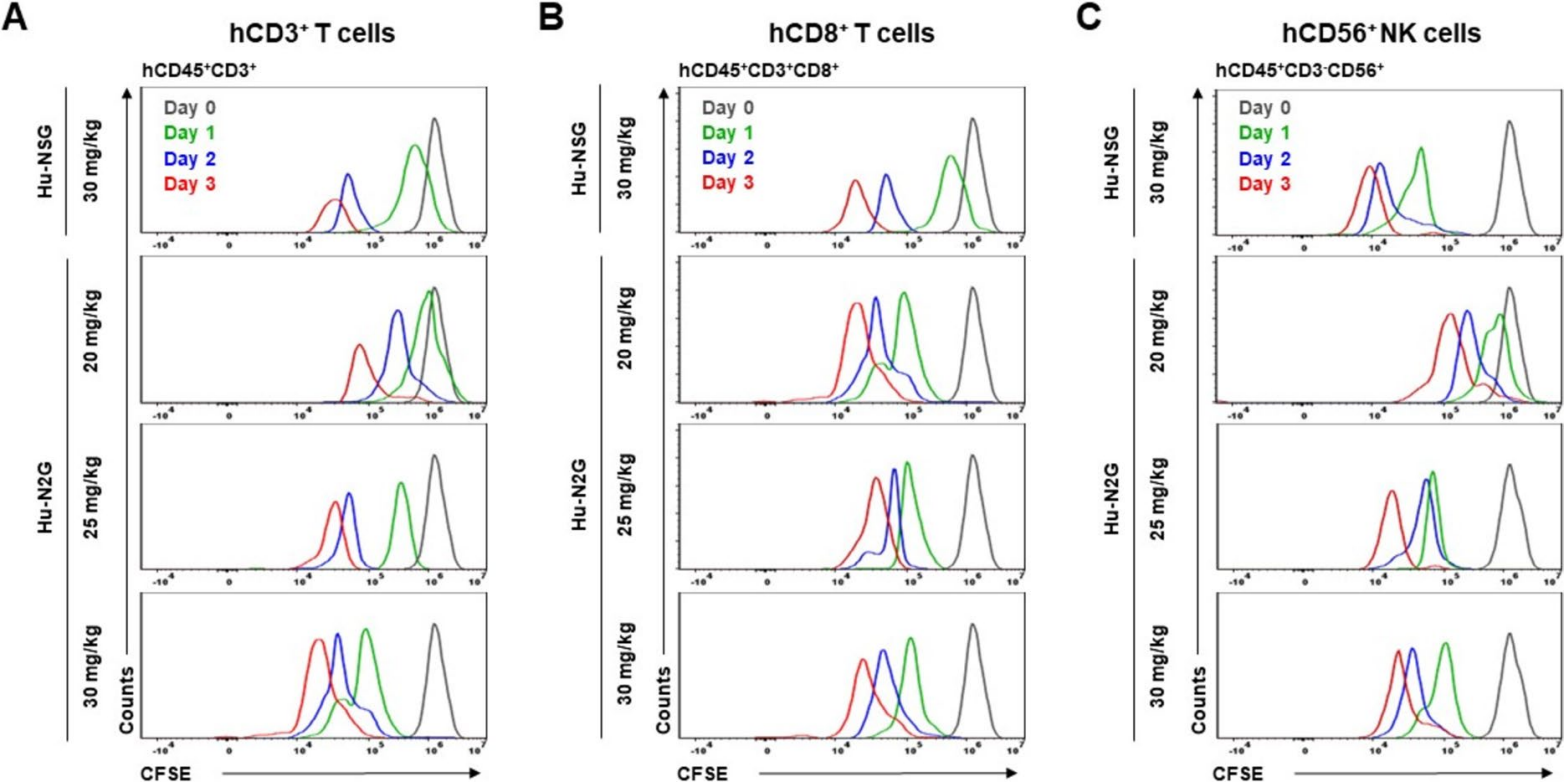
Cell proliferation capacity of splenocytes. **A–C** Splenocytes were isolated from the spleens of hu-N2G (*n* = 3) and hu-NSG (*n* = 3) mice and stained with CFSE dye. Cells were collected daily from day 0 to day 3 and stained with anti-hCD45, anti-hCD3, anti-hCD8, and anti-hCD56 antibodies, followed by flow cytometry analysis

To evaluate the proliferative capacity of splenic T and NK cells in humanized mice, we measured cell division rate using 6-carboxyfluorescein diacetate succinimidyl ester (CFSE) in hCD3+ T cells, hCD8+ T cells, and hCD56+ NK cells. Cells were collected daily from day 0 to day 3, and intracellular cytokine staining was performed to evaluate their proliferation via flow cytometry. Particularly, the proliferation of hCD3+ T cells was significantly higher in hu-N2G mice compared to hu-NSG mice (Fig. 7A). Consistent with this, both hCD8+ T cells and hCD56+ NK cells exhibited robust proliferation in hu-N2G mice (Fig. 7B, C). The proliferation rates of hCD4+ T cells were consistent with these findings (Fig. S6A). Overall, these results demonstrate that reconstituted human CD8+ T and NK cells in hu-N2G mice retain strong proliferation capacity, further supporting the suitability of hu-N2G mice for functional studies of human immune responses.

## Discussion

Given the challenges in evaluating tumors without placing human subjects at risk, there is a growing demand for animal models that enable in vivo studies of human cancers (Kanaji et al. 2014, Morgan 2012). To meet this need, various mouse models have been developed to more accurately recapitulate human tumor biology (Kanaji et al. 2014). Among these, the N2G mouse model represents a notable advancement in preclinical research by addressing some limitations of existing immunodeficient models such as NSG and NOG mice. By achieving near-complete deletion of *Prkdc* and *Il2rg* genes using CRISPR/Cas9 technology, the N2G mice minimize the presence of mutant mRNAs and residual truncated proteins that have been shown to retain partial functionality in current models. As a result, *Prkdc* and *Il2rg* transcripts are undetectable in N2G mice. Notably, extensive deletion of *Prkdc* diminishes the expression of truncated DNA-PKcs, thereby suppressing any kinase-dead variants that could interfere with DNA repair and immune system studies. Likewise, deletion of *Il2rg* ensures the complete absence of non-canonical signaling or residual cytokine receptor activity, which could confound experimental outcomes.

The xenograft experiments using human cancer cell lines (A549 and MDA-MB-231) demonstrated that N2G mice support robust tumor growth, comparable to that observed in NOG mice. Moreover, N2G mice enabled liver metastasis of MDA-MB-231 cells, highlighting their utility in studying cancer progression and metastasis mechanisms. These results indicate that N2G mice provide a reliable and effective platform for human cancer cell xenograft studies, offering a robust alternative to conventional immunodeficient models such as NOG mice for evaluating therapeutic strategies targeting both primary and metastatic tumors.

Furthermore, we successfully achieved engraftment of human immune cells derived from HSCs in N2G mice, with human leukocytes detected in the peripheral blood, spleen, and bone marrow. Notably, hu-N2G-bu30s exhibited significantly improved reconstitution of human hCD3^+^ T and hCD4^+^ T cells in lymphoid organs compared to hu-NSG-bu30s. Moreover, hCD8^+^ T cell populations were significantly increased in the peripheral blood and spleen of hu-N2G-bu30s and exhibited enhanced responsiveness upon stimulation. Although, the proliferation capacity of hCD4^+^ T cells in hu-N2G mice was comparable to that observed in hu-NSG mice, early expansion of hCD3^+^ T and hCD8^+^ T cells was more pronounced in hu-N2G-bu30s than hu-NSG-bu30s. Collectively, these results suggest that N2G mice are well-suited for investigating human immune responses and can serve as a versatile platform for humanized mouse models.

Importantly, the N2G mouse model, with its enhanced immunological profile, offers distinct advantages for translational applications, particularly in the fields of immunotherapy and personalized medicine. The robust reconstitution of functional human T cells—including CD4⁺ and CD8⁺ subsets with superior proliferative capacity, cytokine responsiveness, and cytotoxic activity—together with the development of active NK cells, positions the N2G model as an ideal platform for evaluating adoptive cell therapies such as CAR-T cells (Shultz et al. 2019; Choi et al. 2020; Bulliard et al. 2023). In conventional NSG or NOG models, suboptimal T cell engraftment and functionality have limited in vivo assessment of CAR-T cell dynamics (Kenney et al. 2016; Shultz et al. 2019). By contrast, the improved human T cell repertoire in N2G mice is expected to enable more accurate modeling of CAR-T cell persistence, expansion, and memory differentiation—key determinants of therapeutic efficacy—especially when combined with IL-2 signaling modulation via CAR-enhancer technologies (Rakhshandehroo et al. 2025). Furthermore, the heightened functionality of cytotoxic T cells and NK cells in N2G mice facilitates reliable evaluation of immune checkpoint inhibitors (e.g., anti-PD-1, anti-CTLA-4), particularly in settings requiring robust tumor infiltration and T cell activation (Choi et al. 2020; Shultz et al. 2012; Wang et al. 2024). The near-complete deletion of *Prkdc* and *Il2rg* in N2G mice also eliminates cytokine leakiness and reduces immunological background noise, thereby enhancing the fidelity of immune-oncology studies (Bak et al. 2020; Lee et al. 2020). Additionally, this immunological refinement renders the N2G model highly suitable for evaluating combination therapy strategies—such as CAR-T cells with radiotherapy—where precise temporal control and dose-dependent synergy are critical (Sugita et al. 2023; Lynch et al. 2024). Finally, the N2G model provides a physiologically relevant system for evaluating patient-derived immune cells and/or tumor tissues, addressing key unmet needs in translational research. Collectively, these features position the N2G model as a next-generation platform that bridges the gap between preclinical evaluation and clinical implementation.

Graft-versus-host disease (GVHD) is a condition in which transplanted human immune cells, particularly mature T cells, attack the host tissues, commonly arising in humanized mouse models during long-term engraftment (Sonntag et al. 2015). While we did not observe symptoms of GVHD in either NSG or N2G mice during the 24-week observation period following hCD34⁺ cell transplantation, previous studies have reported the onset of GVHD in NSG mice around 25 weeks post-transplantation (Elhage et al. 2022). Although the N2G line may exhibit altered susceptibility due to the complete deletion of *Il2rg* and *Prkdc*, we did not assess this beyond 24 weeks in the current study. Therefore, future work will be necessary to determine whether GVHD arises in N2G mice during longer-term engraftment periods.

Although busulfan is associated with hematotoxic side effects, including bone marrow failure when misapplied (McCune and Holmberg 2009; Sauer et al. 2007), it remains beneficial in HSC transplantation research due to its lower toxicity compared to TBI (McCune and Holmberg 2009; Ciurea and Andersson 2009). Unlike TBI, busulfan does not necessitate specialized infrastructure or extensive post-treatment care, making it a more accessible option in preclinical setting (Hsieh et al. 2007). Consequently, TBI has been largely supplanted by chemotherapeutic agents such as busulfan for conditioning regimens (Hayakawa et al. 2009). However, dose-dependent variability in hCD34^+^ CB cell engraftment efficiency observed in N2G mice may reflect the hematotoxic effects inherent to busulfan-based conditioning. Despite this, N2G mice effectively support the engraftment, reconstitution, and multilineage differentiation of human immune cells derived from transplanted human CD34^+^ CB cells. Continued optimization of conditioning protocols may further enhance the degree of humanization achievable in this model.

The N2G mouse is poised to serve as a powerful platform for a wide range of biomedical applications and preclinical research. The humanized N2G mouse model enables in vivo investigation of human immune system interactions with cancer cells or tissues and facilitates preclinical evaluation of drug responses in a human immune context. This capacity positions the N2G model as a critical tool for advancing personalized medicine by enabling the assessment of patient-specific therapeutic strategies.

## Supplementary Information

Below is the link to the electronic supplementary material.

Supplementary Figure 1 Genotyping strategy and PCR-based validation of CRISPR/Cas9-mediated deletions in N2G mice. **A** Representative agarose gel electrophoresis results showing PCR amplification of the Prkdc and Il2rg loci from genomic DNA isolated from wild-type (WT) and N2G mice. WT Prkdc produces a 373 bp band, while the CRISPR/Cas9-targeted Prkdc allele in N2G mice yields a 467 bp band. WT Il2rg yields a 247 bp band, whereas the Il2rg deletion allele in N2G mice produces a 299 bp band. Primer binding sites are indicated with arrows in the schematic representations of each locus (JPG 93 KB)

Supplementary Figure 2 Cancer cell-derived xenograft in N2G mice. **A** Representative images and tumor volumes of A549 lung cancer tumors shown in Figure 2B. **B** Representative images of metastatic lung tumors shown in Figure 2C. **C** Representative images and tumor volumes of MDA-MB-231 breast cancer tumors shown in Figure 2D. **D** Representative images of metastatic liver tumor shown in Figure G. **E** Tumor volumes after subcutaneous injection of 5×106 A549 lung cancer cells into 10-week-old male N2G mice (n = 9). **F** Tumor volumes after subcutaneous injection of 8 × 106 SK-BR3 breast cancer cells into 8-week-old female N2G mice (n = 10). **G** Tumor volumes after mammary fat pad injection of 5 × 106 MDA-MB-468 breast cancer cells into 7-week-old female N2G mice (n = 13) (JPG 137 KB)

Supplementary Figure 3 Reconstitution of human cells in peripheral blood with hCD34+ CB cells. **A**–**D** Peripheral blood samples were collected from hu-N2G and hu-NSG mice, and the cells were stained with anti-hCD45 **A**, anti-hCD3 **B**, anti-hCD19 **C**, and anti-hCD33 **D** antibodies. Flow cytometry analysis was performed (JPG 207 KB)

Supplementary Figure 4 Reconstitution of human myeloid cells in peripheral blood of humanized mice. **A** Cells were stained with anti-hCD14, and anti-hCD16 antibodies, followed by flow cytometry analysis. **B** Frequencies and absolute cell numbers were determined through flow cytometric gating. **C** Cells were detected with anti-hCD123, and anti-hCD11c antibodies followed by flow cytometry analysis. **D** Frequencies and absolute cell numbers were obtained by flow cytometric gating. Seven mice were used per group in this figure. Data are presented as the mean ± S.D. for each group of mice. Statistical significance: *p < 0.05, **p < 0.01, ***p < 0.001 (JPG 166 KB)

Supplementary Figure 5 Reconstitution of human cells in the bone marrow of humanized mice. **A** Bone marrow cells were stained with anti-hCD45, anti-hCD3, anti-hCD19, anti-hCD4, and anti-hCD8 antibodies, followed by flow cytometry analysis. **B** Frequencies and absolute cell numbers were obtained by flow cytometric gating. Five mice were used per group in this figure. Data are presented as the mean ± S.D. for each group. Statistical significance: *p < 0.05, **p < 0.01 (JPG 179 KB)

Supplementary Figure 6 Cell proliferation capacity of CD4+ T cells. **A** Splenocytes were isolated from the spleens of hu-N2G (n = 3) and hu-NSG (n = 3) mice. To assess the proliferation ability, cells were stained with CFSE dye. The cells were collected from day 0 to day 3 and subsequently stained with anti-hCD45, anti-hCD3, and anti-hCD4 antibodies, followed by flow cytometry analysis (JPG 84 KB)

## Data Availability

The original contributions presented in the study are included in the article and supplementary Material, further inquiries can be directed to the corresponding authors.
