# Supplementary figures and images for: Generation of NOD SCID mice with near-complete deletions of Il2rg and Prkdc for human cancer and HSC engraftment
Source: Transgenic Res. 2025 Jul 11;34(1):35. doi: 10.1007/s11248-025-00454-9 (PMC12254168; doi:10.1007/s11248-025-00454-9)

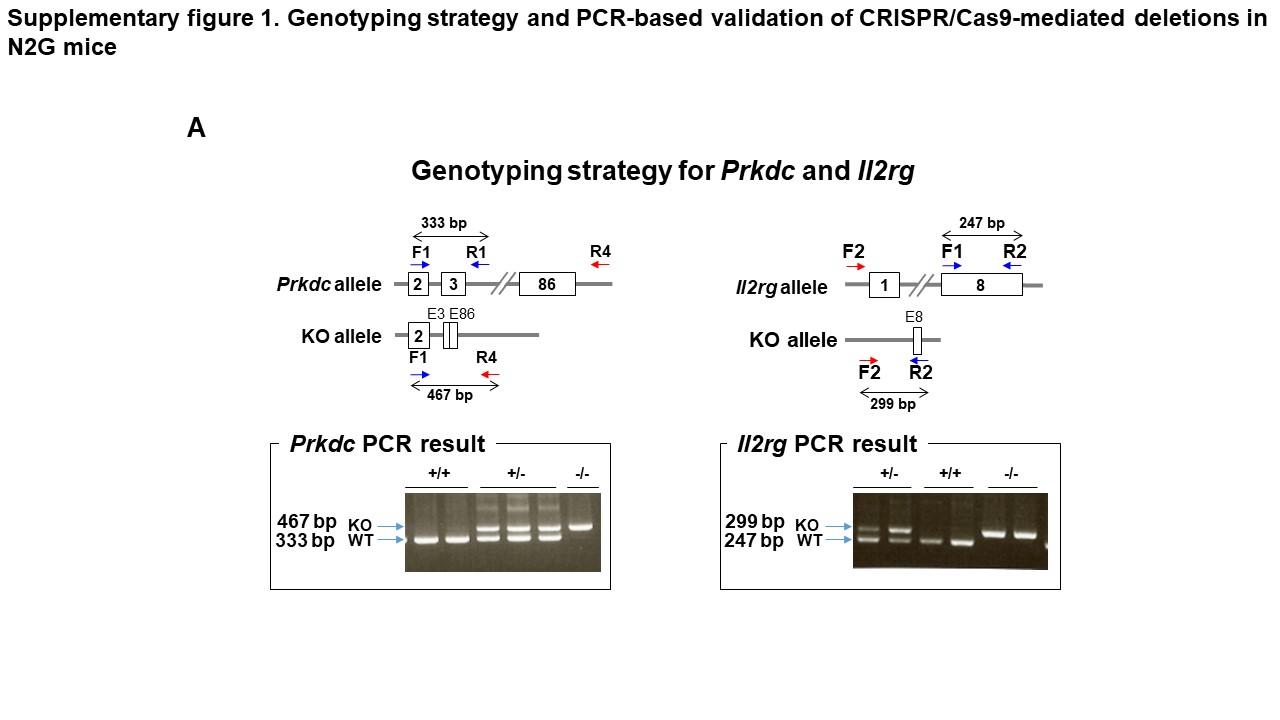

Supplement: Supplementary file 1 — Supplementary Figure 1 Genotyping strategy and PCR-based validation of CRISPR/Cas9-mediated deletions in N2G mice. A Representative agarose gel electrophoresis results showing PCR amplification of the Prkdc and Il2rg loci from genomic DNA isolated from wild-type (WT) and N2G mice. WT Prkdc produces a 373 bp band, while the CRISPR/Cas9-targeted Prkdc allele in N2G mice yields a 467 bp band. WT Il2rg yields a 247 bp band, whereas the Il2rg deletion allele in N2G mice produces a 299 bp band. Primer binding sites are indicated with arrows in the schematic representations of each locus (JPG 93 KB) [file 11248_2025_454_MOESM1_ESM.jpg]

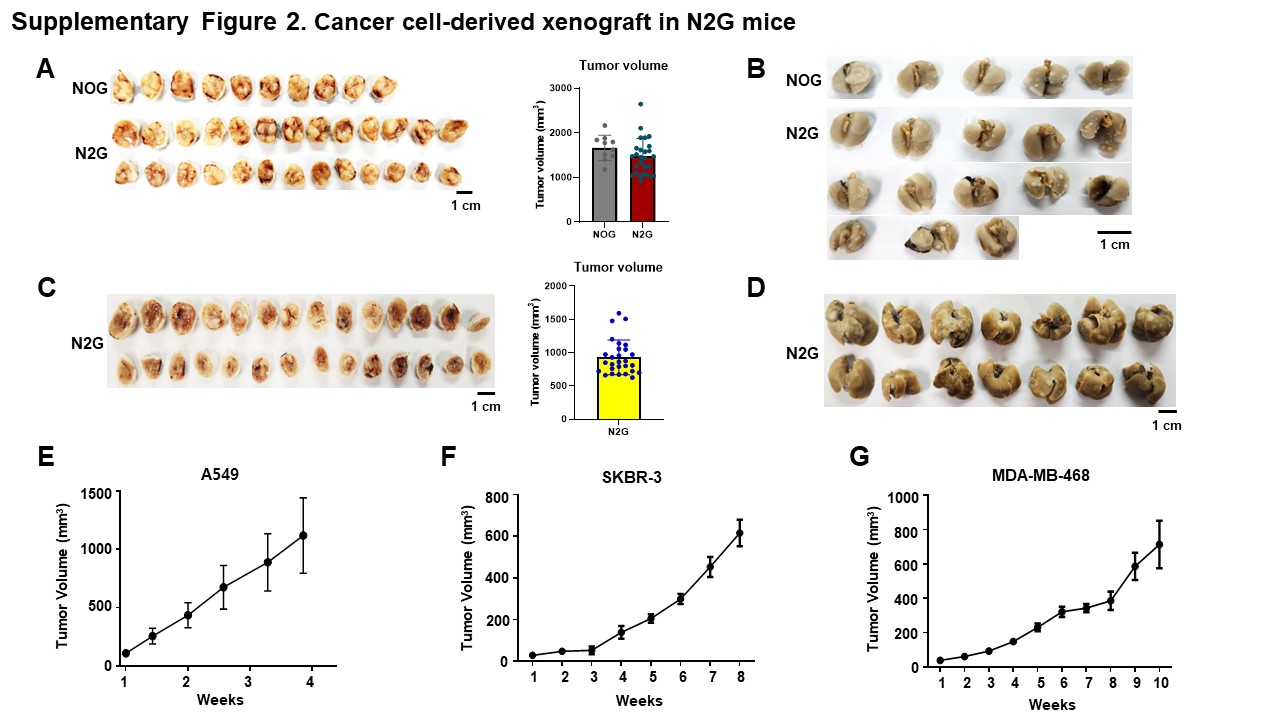

Supplement: Supplementary file 2 — Supplementary Figure 2 Cancer cell-derived xenograft in N2G mice. A Representative images and tumor volumes of A549 lung cancer tumors shown in Figure 2B. B Representative images of metastatic lung tumors shown in Figure 2C. C Representative images and tumor volumes of MDA-MB-231 breast cancer tumors shown in Figure 2D. D Representative images of metastatic liver tumor shown in Figure G. E Tumor volumes after subcutaneous injection of 5×10^6^ A549 lung cancer cells into 10-week-old male N2G mice (n = 9). F Tumor volumes after subcutaneous injection of 8 × 10^6^ SK-BR3 breast cancer cells into 8-week-old female N2G mice (n = 10). G Tumor volumes after mammary fat pad injection of 5 × 10^6^ MDA-MB-468 breast cancer cells into 7-week-old female N2G mice (n = 13) (JPG 137 KB) [file 11248_2025_454_MOESM2_ESM.jpg]

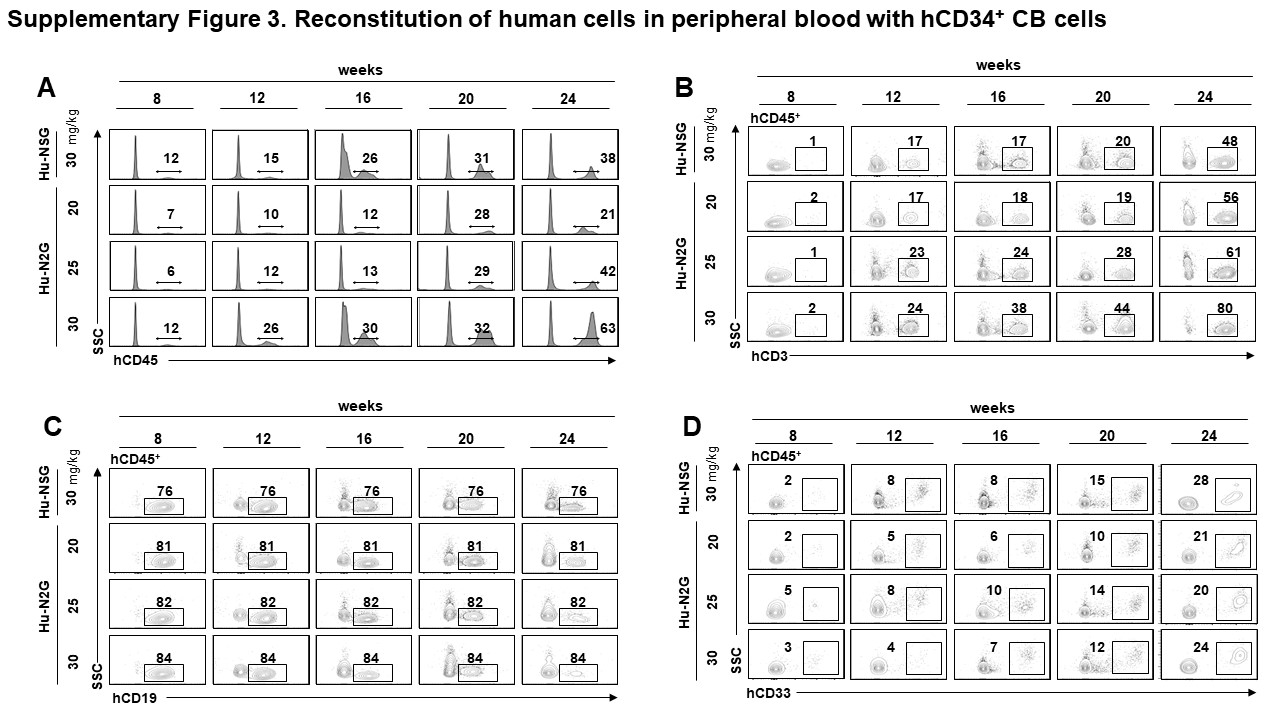

Supplement: Supplementary file 3 — Supplementary Figure 3 Reconstitution of human cells in peripheral blood with hCD34+ CB cells. A–D Peripheral blood samples were collected from hu-N2G and hu-NSG mice, and the cells were stained with anti-hCD45 A, anti-hCD3 B, anti-hCD19 C, and anti-hCD33 D antibodies. Flow cytometry analysis was performed (JPG 207 KB) [file 11248_2025_454_MOESM3_ESM.jpg]

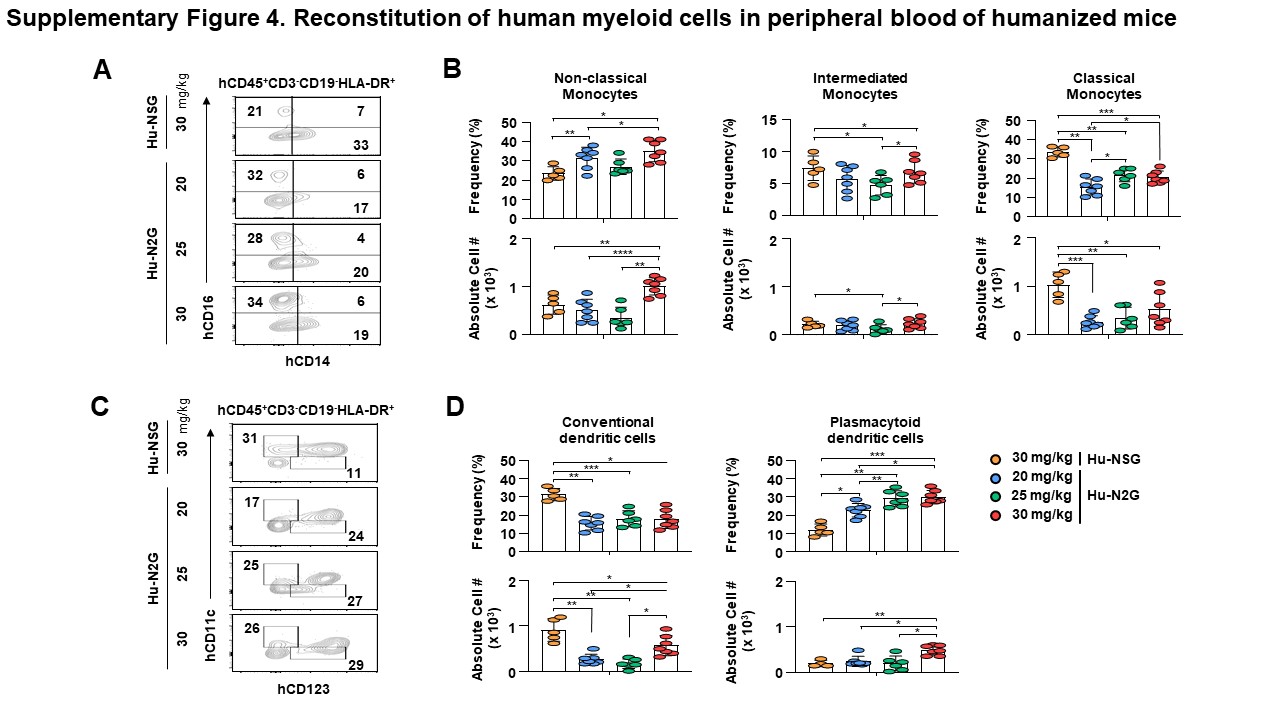

Supplement: Supplementary file 4 — Supplementary Figure 4 Reconstitution of human myeloid cells in peripheral blood of humanized mice. A Cells were stained with anti-hCD14, and anti-hCD16 antibodies, followed by flow cytometry analysis. B Frequencies and absolute cell numbers were determined through flow cytometric gating. C Cells were detected with anti-hCD123, and anti-hCD11c antibodies followed by flow cytometry analysis. D Frequencies and absolute cell numbers were obtained by flow cytometric gating. Seven mice were used per group in this figure. Data are presented as the mean ± S.D. for each group of mice. Statistical significance: *p < 0.05, **p < 0.01, ***p < 0.001 (JPG 166 KB) [file 11248_2025_454_MOESM4_ESM.jpg]

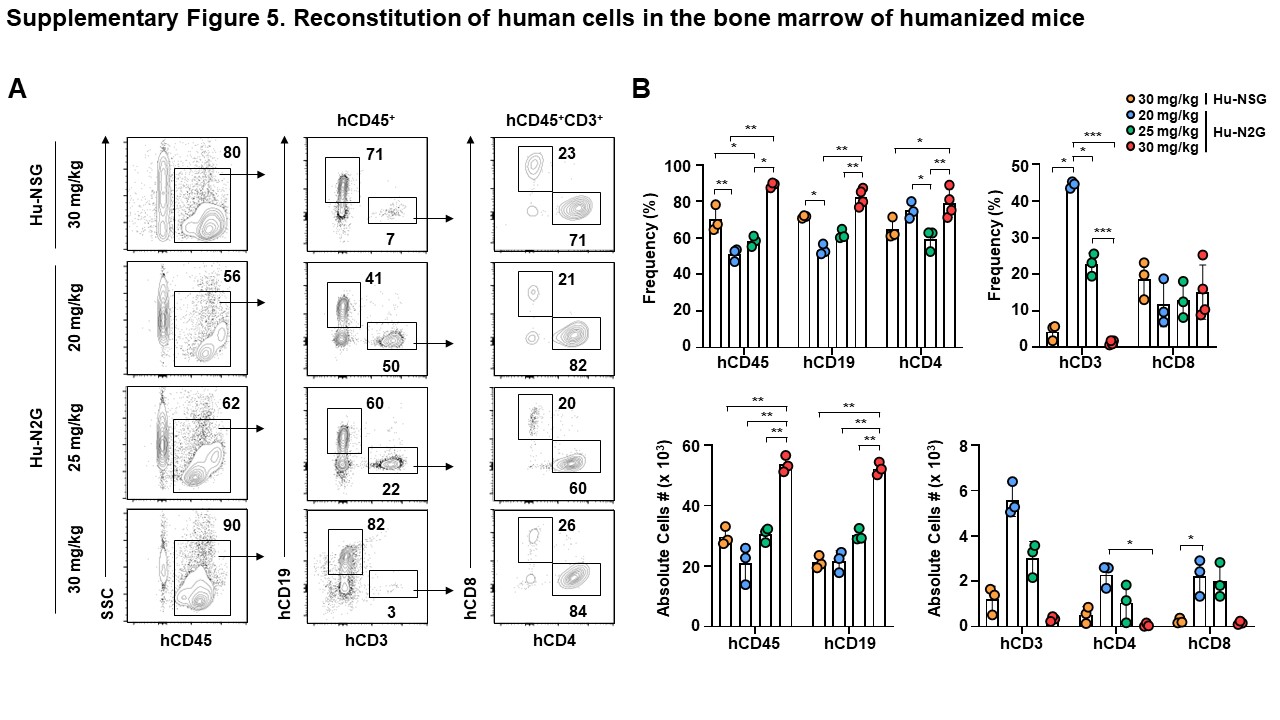

Supplement: Supplementary file 5 — Supplementary Figure 5 Reconstitution of human cells in the bone marrow of humanized mice. A Bone marrow cells were stained with anti-hCD45, anti-hCD3, anti-hCD19, anti-hCD4, and anti-hCD8 antibodies, followed by flow cytometry analysis. B Frequencies and absolute cell numbers were obtained by flow cytometric gating. Five mice were used per group in this figure. Data are presented as the mean ± S.D. for each group. Statistical significance: *p < 0.05, **p < 0.01 (JPG 179 KB) [file 11248_2025_454_MOESM5_ESM.jpg]

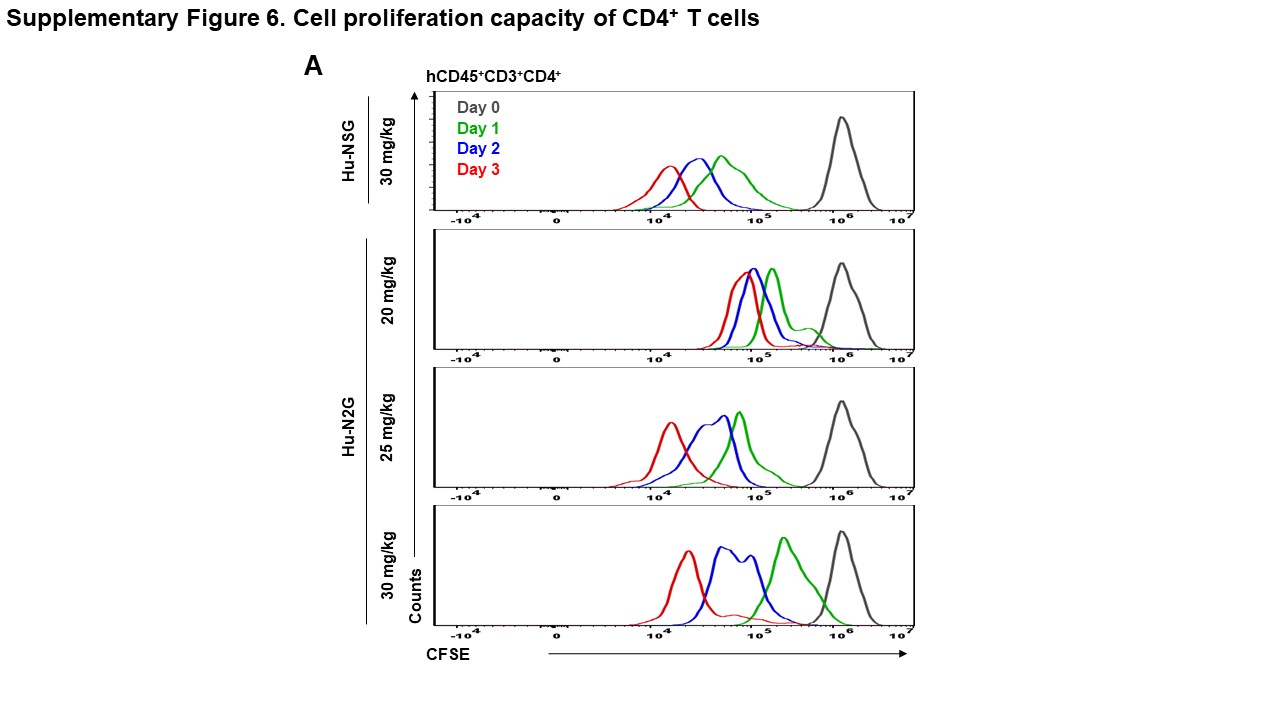

Supplement: Supplementary file 6 — Supplementary Figure 6 Cell proliferation capacity of CD4+ T cells. A Splenocytes were isolated from the spleens of hu-N2G (n = 3) and hu-NSG (n = 3) mice. To assess the proliferation ability, cells were stained with CFSE dye. The cells were collected from day 0 to day 3 and subsequently stained with anti-hCD45, anti-hCD3, and anti-hCD4 antibodies, followed by flow cytometry analysis (JPG 84 KB) [file 11248_2025_454_MOESM6_ESM.jpg]
